# Estrogen-Dependent Proteolytic Cleavage of Semaphorin 4D and Plexin-B1 Enhances Semaphorin 4D-Induced Apoptosis during Postnatal Vaginal Remodeling in Pubescent Mice

**DOI:** 10.1371/journal.pone.0097909

**Published:** 2014-05-19

**Authors:** Takuji Ito, Tao Bai, Tetsuji Tanaka, Kenji Yoshida, Takashi Ueyama, Masayasu Miyajima, Takayuki Negishi, Takahiko Kawasaki, Hyota Takamatsu, Hitoshi Kikutani, Atsushi Kumanogoh, Kazunori Yukawa

**Affiliations:** 1 Department of Physiology, Faculty of Pharmacy, Meijo University, Nagoya, Japan; 2 Department of Obstetrics and Gynecology, Wakayama Medical University, Wakayama, Japan; 3 Department of Anatomy and Cell Biology, Wakayama Medical University, Wakayama, Japan; 4 Laboratory Animal Center, Wakayama Medical University, Wakayama, Japan; 5 Division of Brain Function, National Institute of Genetics, Graduate University for Advanced Studies (Sokendai), Mishima, Japan; 6 Department of Immunopathology, Research Institute for Microbial Diseases, Osaka University, Suita, Japan; 7 Department of Molecular Immunology, Research Institute for Microbial Diseases, Osaka University, Suita, Japan; Toho University School of Medicine, Japan

## Abstract

Around the fifth week after birth, the vaginal cavity in female mouse pups opens to the overlaying skin. This postnatal tissue remodeling of the genital tract occurs during puberty, and it largely depends upon hormonally induced apoptosis that mainly occurs in the epithelium at the lower part of the mouse vaginal cavity. Previously, we showed that most BALB/c mice lacking the class IV Semaphorin (Sema4D) develop imperforate vagina and hydrometrocolpos; therefore, we reasoned that the absence of Sema4D-induced apoptosis in vaginal epithelial cells may cause the imperforate vagina. Sema4D signals via the Plexin-B1 receptor; nevertheless detailed mechanisms mediating this hormonally triggered apoptosis are not fully documented. To investigate the estrogen-dependent control of Sema4D signaling during the apoptosis responsible for mouse vaginal opening, we examined structural and functional modulation of Sema4D, Plexin-B1, and signaling molecules by analyzing both wild-type and Sema4D−/− mice with or without ovariectomy. Both the release of soluble Sema4D and the conversion of Plexin-B1 by proteolytic processing in vaginal tissue peaked 5 weeks after birth of wild-type BALB/c mice at the time of vaginal opening. Estrogen supplementation of ovariectomized wild-type mice revealed that both the release of soluble Sema4D and the conversion of Plexin-B1 into an active form were estrogen-dependent and concordant with apoptosis. Estrogen supplementation of ovariectomized Sema4D−/− mice did not induce massive vaginal apoptosis in 5-week-old mice; therefore, Sema4D may be an essential apoptosis-inducing ligand that acts downstream of estrogen action in vaginal epithelium during this postnatal tissue remodeling. Analysis of ovariectomized mice also indicated that Sema4D contributed to estrogen-dependent dephosphorylation of Akt and ERK at the time of vaginal opening. Based on our results, we propose that apoptosis in vaginal epithelium during postnatal vaginal opening is induced by enhanced Sema4D signaling that is caused by estrogen-dependent structural changes of Sema4D and Plexin-B1.

## Introduction

In mice, the blind ending of the vaginal cavity in each female pup opens to the skin around 5 weeks after birth when sex hormone level rises in the internal environment; vaginal opening is one of very few postnatal tissue remodeling events in mice [1]. The study of transgenic mice that overexpress the human anti-apoptotic protein Bcl-2 in the vaginal mucosa clearly shows that this postnatal tissue remodeling process depends heavily on massive mucosal apoptosis; these cell deaths occur in a very limited time window and only at the lower distal end of mouse vaginal cavity in the vicinity of skin [1]. Subsequently, several studies involving various types of knockout mice revealed the crucial involvement of proapoptotic Bcl2 family proteins [2], [3], along with other signaling molecules, in mouse vaginal remodeling [4], [5]. However, the exact mechanisms by which rapid increases of estrogen level in the mouse internal environment induce the extensive apoptosis in vaginal epithelium at the time of puberty remain unknown [1], [6]. We found that mice lacking Semaphorin 4D (Sema4D) often develop imperforate vagina and hydrometrocolpos [7]; Sema4D is a semaphorin that controls axon guidance during neuronal development [8], [9].

Semaphorins are a family of secreted and transmembrane glycoproteins with phylogenetically conserved domains; they were originally identified as repulsive axon guidance molecules that function during development of the nervous system [10]. Sema4D (also called CD100) is a class 4 transmembrane-type semaphorin that binds to Plexin-B1, which is a transmembrane receptor and a member of the plexin family, to induce repulsive cytoskeletal changes in growth cones of cultured neurons [11]. Sema4D and Plexin-B1 interact via their conserved sema domains of ∼400 amino acids each; each sema domain forms a seven-blade β-propeller fold in the extracellular domain of the respective protein [10], [12], [13]. Binding of Sema4D to Plexin-B1 causes Plexin-B1 molecules to cluster; this clustering facilitates GTPase activating protein (GAP) activities of the two GAP domains in the intracellular region of each Plexin-B1 molecule [14]. The augmented Plexin-B1 GAP activities in neurons 1) downregulate activities of Ras family members and 2) induce dephosphorylation of Akt and ERK and activation of glycogen synthase kinase (GSK)-3β; these events reduce integrin-mediated cell adhesion to the extracellular matrix, and consequently induce morphological remodeling of growth cones and dendrites in cultured neurons [14], [15], [16]. In contrast, Sema4D binding to Plexin-B1 on endothelial cells stimulates the activation of the phosphatidylinositol 3-kinase (PI3K)-Akt pathway and of ERK to induce endothelial cell migration [17], [18]. Thus the Sema4D signal either facilitates or weakens the same intracellular signaling pathway depending upon the cell type and cellular context. Membrane-bound Sema4D on T lymphocytes is cleaved via metalloprotease-dependent proteolysis, and the extracellular domain is then shed as diffusible secreted protein from a membrane surface to the surrounding extracellular environment [19]. Soluble secreted Sema4D is thought to function as a guidance cue that acts across long distances to inhibit immune cell migration [19]. Plexin-B1 is also converted from a precursor into an active heterodimeric form composed of distinct subunits resulting from proprotein convertase-mediated processing of Plexin-B1 [20]. The proteolytically processed, active form of Plexin-B1 transmits a more intense Sema4D-dependent intracellular signal than does the unprocessed precursor [20].

The high frequency of vaginal atresia and the significant attenuation of vaginal epithelial apoptosis in Sema4D-deficient (Sema4D−/−) mice indicates that vaginal mucosal apoptosis and the vaginal opening process do not occur normally in these mice. Furthermore, we demonstrated that Sema4D binds to Plexin-B1 receptor to induce apoptosis of vaginal epithelial cells in culture. β-Estradiol administration in infant Sema4D-deficient mice does not induce precocious vaginal opening; therefore, Sema4D may function downstream of estrogen action during postnatal vaginal tissue remodeling [7]. However, mouse vaginal opening occurs 5 weeks after birth when estrogen levels increase rapidly in female mice, and the pro-apoptotic signals in the maturing vaginal epithelial cells may be enhanced via estrogen-mediated functional modulation of Sema4D and Plexin-B1. Here, we examined whether estrogen induces structural and functional changes in Sema4D and Plexin-B1 that lead to the induction of vaginal epithelial apoptosis and the consequent tissue remodeling. The results of our study indicated 1) that Sema4D cleavage and Plexin-B1 activation were both estrogen dependent and 2) that these events led to vaginal epithelial apoptosis in this postnatal tissue remodeling event.

## Materials and Methods

### Generation of Sema4D−/− Mice

Sema4D−/− mice generated by gene targeting [21] were backcrossed with BALB/c mice for 10 generations. Pairs of resultant heterozygous mice were bred to obtain homozygous, knockout mice and their wild-type (WT) littermates. The mice were bred for the preparatory and pairwise in the animal facilities of Wakayama Medical University and the animal center in the Faculty of Pharmacy, Meijo University. Each researcher and each laboratory technician followed the guidelines promulgated by the Physiological Society of Japan and the respective guidelines on animal experiments from Wakayama Medical University and from Meijo University when caring for or sacrificing mice and when conducting protocols involving mice. Each institutional Animal Ethics Review committee, the Wakayama Medical University committee and the Meijo University committee, approved the experimental protocols (approval number: 267; Wakayama Medical University, 2012-yaku-jitsu-8; Meijo University).

### Genotype Analysis

Mouse tail DNA, Sema4D gene-specific primers, and PCR were used as described previously to determine each mouse genotype [21].

### Immunohistochemistry and TUNEL Assay

Mice were sacrificed by intraperitoneal injection of pentobarbital sodium (Kyoritsuseiyaku Co., Tokyo, Japan) and then subjected to transcardiac perfusion of 4% paraformaldehyde. Each vagina excised from a mouse was fixed overnight in 4% paraformaldehyde solution. Each vagina was then embedded longitudinally in paraffin and cut into 4-µm serial sections. Sections were immunolabeled with anti-cleaved caspase-3 (Cell Signaling Technology, Beverly, MA), anti-Akt (Cell Signaling Technology), or anti-Erk1/2 antibody (Cell Signaling Technology). TUNEL assays were performed basically as described previously [22], using a fluorometric TUNEL assay system (Promega, Madison, WI) following the manufacturer’s protocols.

### Western Blot

To reduce the variance among samples, dissection of each mouse vagina was conducted as follows. A mouse was sacrificed via intraperitoneal injection of pentobarbital sodium; a laparotomy was then performed to expose the reproductive organs, and the fatty tissues around the vagina were then removed. The pubic bone was resected to view the lower region of the vagina; the urinary bladder, urethra, and rectum were then separated from the vagina. In each case of an unopened vagina, the border area between the lowest end of the vagina and the skin surface of the expected vaginal orifice was transversely cut so that the septum covering the lower extremity of the vagina [1] was included in the vaginal tissue sample; simultaneously, the uterine cervix was pulled ventrally with forceps. In each case of an opened vagina, the region between the vaginal orifice and the surrounding skin was cut while the uterine cervix was pulled ventrally with forceps. Each vagina was obtained via a transverse cut beneath the lowest extremity of the uterine cervix to exclude the cervix; each such vagina was rapidly minced into small pieces on ice. Tissue extracts were prepared by homogenizing the mouse vaginal tissue in T-PER Tissue Protein Extraction Reagent (Thermo Scientific Inc., Waltham, MA) with a protease inhibitor, α-complete (Roche Applied Science, Penzberg, Germany), and a phosphatase inhibitor, PhosStop (Roche Applied Science). The Bio-Rad Protein assay (Bio-Rad, Hercules, CA) was used to measure the protein content in each tissue extract, and samples containing 15 µg of protein were prepared with a solution of 60 mM Tris-HCl (pH 6.8), 2% SDS, 10% glycerol, 0.1% bromophenol blue, and 5% β-mercaptoethanol; each of these samples was incubated at 100°C for 5 min and subjected to electrophoresis through a 10% SDS-polyacrylamide gel; separated proteins were then transferred to polyvinylidine difluoride membranes (Amersham Pharmacia Biotech, Buckinghamshire, UK). Sema4D, plexin-B1, cleaved caspase-3, Akt, phospho-Akt, ERK1/2, and phospho-ERK1/2 were detected with the respective antibodies and an ECL-plus or ECL Western blot detection system in accordance with the manufacturers’ instructions (Amersham). The antibodies utilized were anti-CD100/Sema4D (BD Transduction Laboratories, NJ, USA), anti-plexin-B1 (Santa Cruz Biotechnology, Inc.), anti-cleaved caspase-3 (Cell Signaling Technology), anti-Akt (Cell Signaling Technology), anti-phospho-Akt (Cell Signaling Technology), anti-Erk1/2 (Cell Signaling Technology), anti-phospho-Erk1/2 antibody (Cell Signaling Technology), and β-actin antibody (Cell Signaling Technology).

### Ovariectomy and Estrogen Supplementation

For each ovariectomy (OVX), both ovaries were excised from a 22-day-old mouse while the mouse was under anesthesia from intraperitoneal injection of pentobarbital sodium. As controls, sham operations, in which both ovaries were manipulated but not resected, were performed on WT or Sema4D−/− mice. Ovariectomized mice were then separated into three groups: in two groups, from postnatal day 24 to postnatal day 34, β-estradiol (E2, 0.1 µg/kg body weight, Sigma Chemical Co., St. Louis, MO) (OVX-E2 group) or vehicle oil (OVX-oil group) was injected daily and subcutaneously into each mouse, and in the remaining group, no injection was administered (OVX group). The OVX-E2 and OVX-oil groups were sacrificed for Western and immunohistochemical analysis 24 hours after the last injection. Both sham-operated and OVX mice were also sacrificed for Western and immunohistochemical analysis on postnatal day 35. Uterine weight of each mouse was measured to assess the effects of OVX, oil supplementation (OVX-oil) and estrogen supplementation (OVX-E2), as shown in Fig. S1.

### Real-time RT-PCR

The SV Total RNA Isolation System (Promega, Madison, WI) was used according to the manufacturer’s instructions to extract RNA from vaginal tissues of sham-operated WT and Sema4D−/− mice, as well as from OVX, OVX-oil, and OVX-E2 mice. Total RNA was extracted from developing vaginal tissues via the same procedure. The QuantiTect Primer Assays and the following probes Mm_Sema4d_1_SGQT00115206, Mm_Plxnb1_1_SG QT00126483, Mm_B2m_2_SG QT01149547 (Qiagen, Tokyo, Japan) were used according to the manufacturer’s instructions to perform real-time RT-PCR.

### Statistics

Each data value was expressed as a mean ± standard error of the mean (SEM). Comparisons between WT and Sema4D−/− mice were conducted with the Student’s t-test or a one-way or two-way analysis of variance (ANOVA) followed by a *post hoc* test. A level of p<0.05 was considered statistically significant.

## Results

### Conversion of Membrane-bound Sema4D to Soluble Secreted Sema4D during Vaginal Development

**Figure 1:**
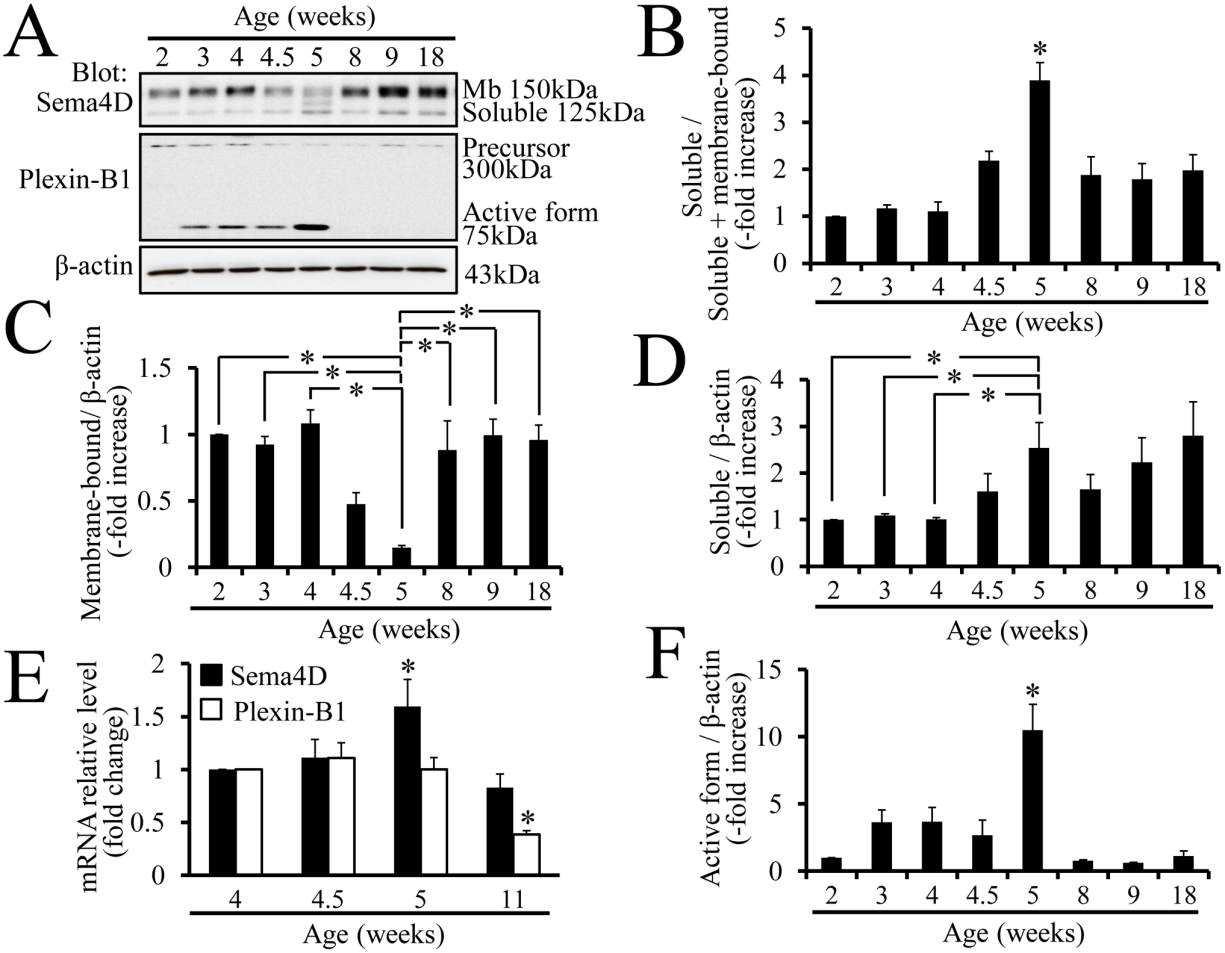
Increase of soluble Sema4D and reorganization of Plexin-B1 receptor during vaginal development. (A) The membrane-bound (larger) and soluble (smaller) forms of Sema4D are evident on western blots containing proteins from vaginal tissue extracts. The precursor (larger) and active form (smaller) of Plexin-B1 are also evident on western blots containing proteins from vaginal tissue extracts; the active 75 kDa form represents a fragment of the 300 kDa Plexin-B1 precursor produced by proprotein convertase. The fragments resulting from the proprotein convertase-dependent cleavage are integrated into signaling-active receptors, which have a distinct conformation from the precursors [20]. Age (weeks): vaginal protein extracts from 2-, 3-, 4-, 4.5-, 5-, 8-, 9-, or 18-week-old mice. Mb: membrane-bound. (B) The ratio of soluble Sema4D to total Sema4D increases significantly in 5-week-old mouse vaginal tissue. Each data point represents the mean ± SEM of 3 to 6 mice. *P<0.05, ANOVA. (C) The ratio of membrane-bound Sema4D to β-actin significantly decreases in 5-week-old vaginal tissue. Each column represents the mean ± SEM of 4 mice. *P<0.05, ANOVA. (D) The ratio of soluble Sema4D to β-actin in 5-week-old mouse vaginal tissue is significantly higher than that in 2-, 3-, or 4-week-old mouse vaginal tissue. Each value represents the mean ± SEM of 3 to 6 mice. *P<0.05, ANOVA. (E) Based on real-time PCR data, the expression of Sema4D mRNA increases significantly in vaginal tissue from 5-week-old mice as compared with that from any other developmental stage. Plexin-B1 mRNA levels show no changes during vaginal opening except for a significant decline in 11-week-old mice. Each column represents the mean ± SEM of 4 mice. *P<0.05, ANOVA. (F) The ratio of active Plexin-B1 to β-actin is significantly higher in 5-week-old mouse vaginal tissue than in that from any other developmental stage. Each point represents the mean ± SEM of 3 to 6 mice. *P<0.05, ANOVA.

Western blots were probed with anti-Sema4D antibodies to examine the expression patterns of membrane-bound and soluble secreted Sema4D during vaginal development; samples of tissue extracts were taken from each stage of postnatal mouse vaginal development. The ratio of soluble to total Sema4D was significantly higher 5 weeks after birth, which is when vaginal opening occurs, than at any other developmental stage (Fig. 1A, B). Membrane-bound Sema4D level was significantly lower at 5 weeks after birth than at any other developmental stage (Fig. 1C). The results illustrated that the rate of conversion from membrane-bound Sema4D to secreted Sem4D was significantly higher at the time of vaginal opening than at any other developmental stage.

Concordant with the significantly higher level of soluble secreted Sema4D in 5-week-old vaginal tissues, the *Sema4D* mRNA level was also significantly higher in 5-week-old vagina tissue based on real-time RT-PCR analysis (Fig. 1E).

### Reorganization of Plexin-B1 during Vaginal Development

Western blots were probed with anti-Plexin-B1 antibodies to examine whether the expression of the Sema4D receptor, Plexin-B1, changed during vaginal development. Vaginal tissue protein extracts from each developmental stage and anti-Plexin-B1 antibodies that detected both a 300 kDa form and a smaller 75 kDa form were used for this analysis (Fig. 1A). The 300 kDa protein represents the precursor form of Plexin-B1 that exists prior to digestion by a protease, proprotein convertase [20]. The smaller 75 kDa protein represents the Plexin-B1 fragment that is generated by proprotein convertase-mediated digestion [20]. After convertase-mediated digestion of the Plexin-B1 precursor, the conformation of Plexin-B1 protein structure is transformed into an active form that transmits Sema4D signal more intensely (20, Fig. S2). Interestingly, the ratio of the smaller 75 kDa band to the β-actin band was significantly higher for the 5-week sample than for any other sample (Fig. 1F). Thus the western blot findings indicated that the active form of Plexin-B1 was highest at 5 weeks, which is the time of mouse vaginal opening. Real-time PCR analysis demonstrated that *Plexin-B1* mRNA levels were constant during postnatal vaginal development except that the mRNA declined significantly by the 11th week (Fig. 1E).

### Conversion of Sema4D and of Plexin-B1 is Estrogen-dependent

**Figure 2:**
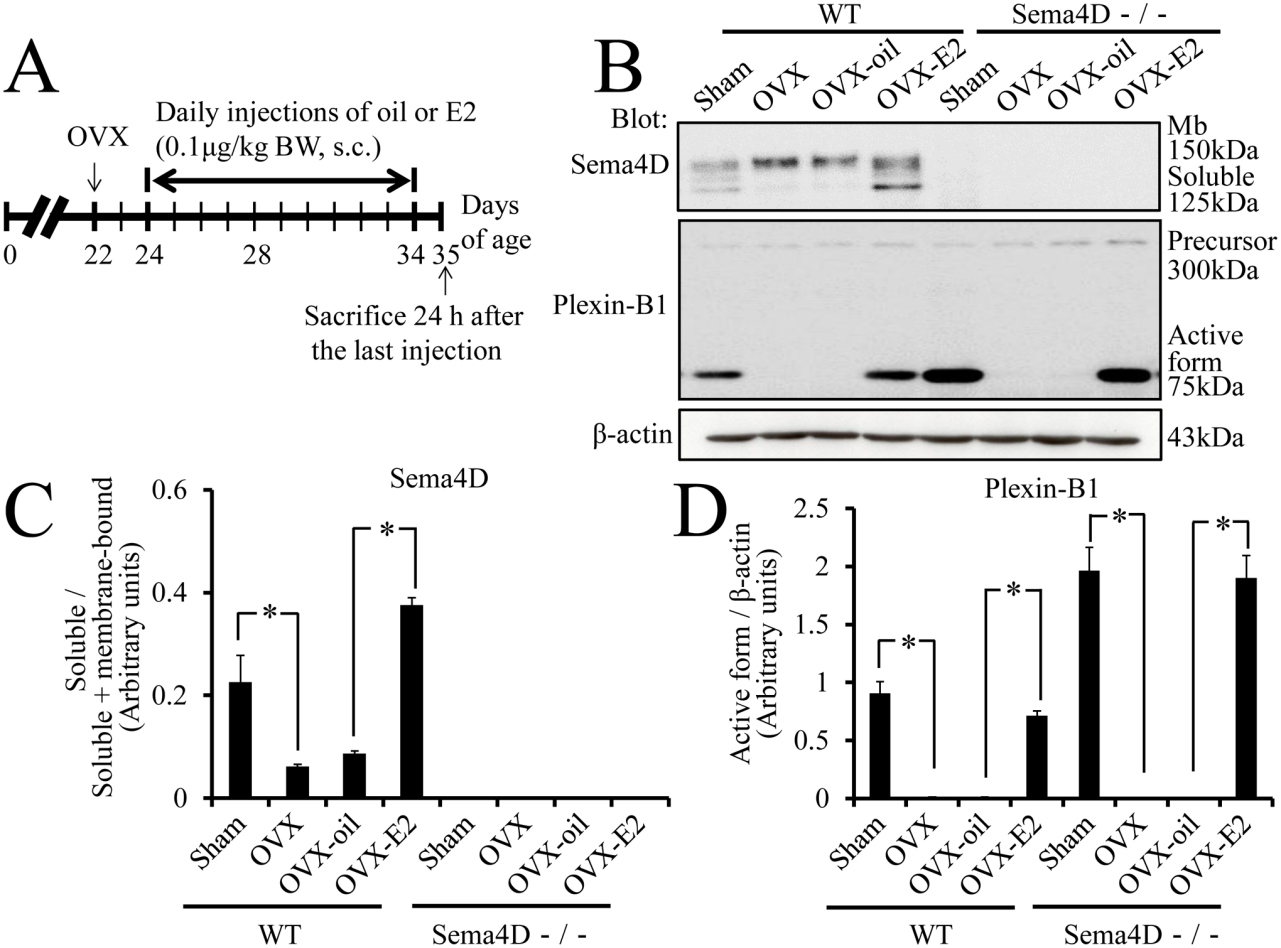
Hormonal regulation of structural changes in Sema4D and Plexin-B1. (A–D) To investigate whether estrogen induces structural changes in Sema4D, Plexin-B1, or both, ovariectomized mice receive daily subcutaneous injections (s.c.) of 17β-estradiol (E2, 0.1 µg/kg) until 5 weeks after birth. As expected, both soluble Sema4D and active Plexin-B1 are detected in vaginal tissue from sham-operated 5-week-old female WT mice (Sema4D+/+). Interestingly, after ovariectomy (OVX) and ovariectomy plus oil supplementation (OVX-oil), the amounts of both soluble Sema4D and active Plexin-B1 significantly decrease. The decrease in active protein levels is rescued by daily subcutaneous injection of E2 (OVX-E2); these findings indicate that the enzymes cleaving membrane-type Sema4D and Plexin-B1 precursor are induced by estrogen. In Sema4D−/− mice, OVX and OVX-oil result in significant decreases in the active form of Plexin-B1; these decreases are abrogated by OVX-E2 treatment. Both membrane-bound and soluble Sema4D are not evident in samples from Sema4D−/− mice. Each value in the graphs (C, D) represents the mean ± SEM of 5 mice. *P<0.05, ANOVA.

Postnatal vaginal remodeling in mice is a hormonally triggered process [1]; therefore, we investigated whether proteolytic release of Sema4D and reorganization of Plexin-B1 during remodeling were each estrogen dependent. Mice were initially subjected to ovariectomy on postnatal day 22; one third of these mice were each injected with exogenous estrogen (OVX-E2 mice), another third with vehicle only (OVX-oil mice, Fig. 2A) and the remaining third received no injection (OVX mice). Western blot analysis was used to examine both Sema4D proteolysis and Plexin-B1 conversion in these ovariectomized mice when the mice became 5 weeks old (Fig. 2B). Conversion of membrane-bound Sema4D into the soluble form was significantly lower in OVX mice than in sham-operated WT mice (Fig. 2B, C). In contrast, this Sema4D conversion was significantly higher in WT OVX-E2 than in WT OVX-oil mice (Fig. 2B, C); these findings indicated that proteolytic conversion of Sema4D was estrogen dependent. Reorganization of Plexin-B1 into an active form was significantly lower in WT OVX mice than in sham-operated WT mice (Fig. 2B, D). The reorganization of Plexin-B1 was significantly higher in WT OVX-E2 mice than in WT OVX-oil mice; these findings indicated Plexin-B1 reorganization during mouse vaginal tissue remodeling was estrogen dependent (Fig. 2B, D). The phenomenon was also confirmed in Sema4D−/− mice (Fig. 2B, D).

**Figure 3:**
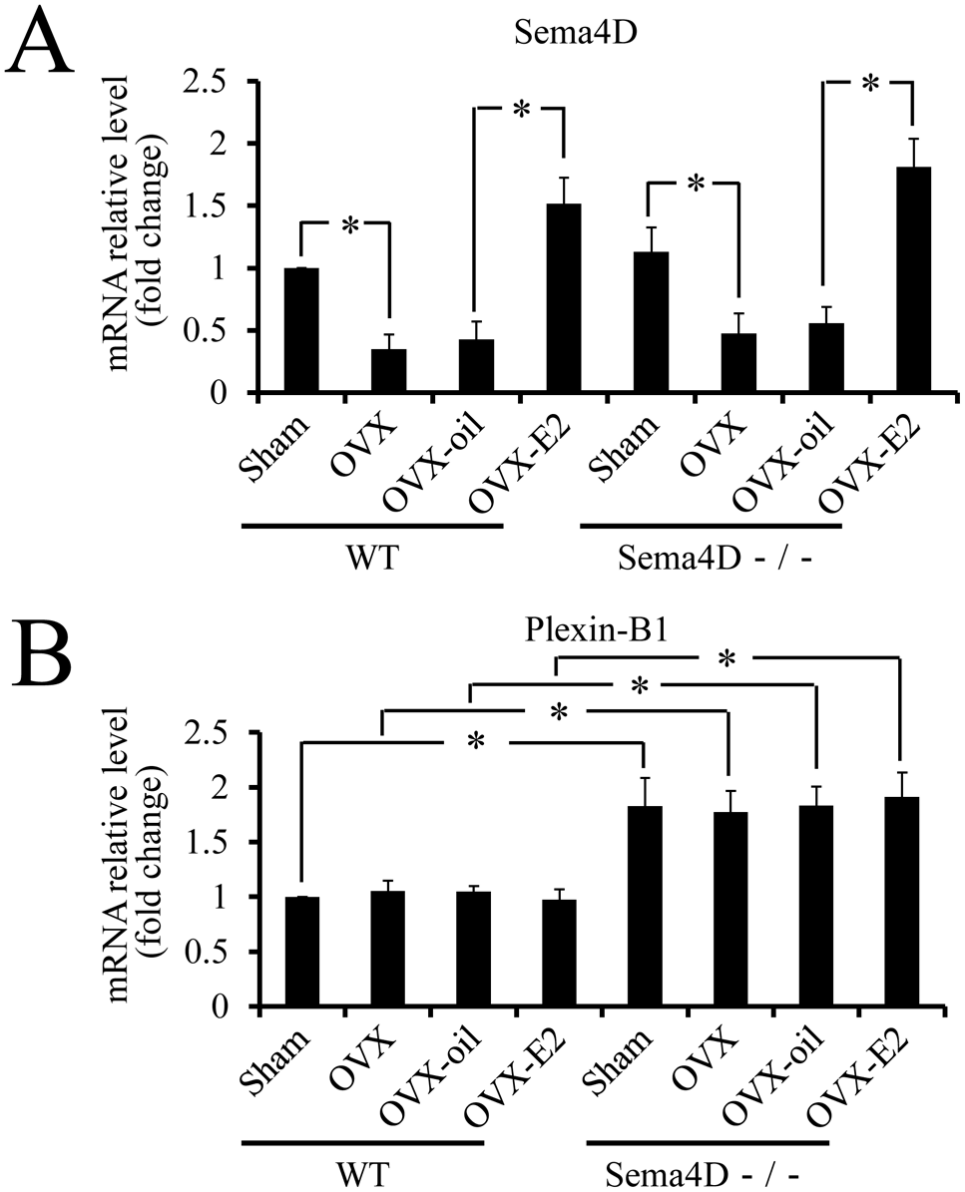
Estrogen increases levels of *Sema4D* mRNA, but not *Plexin-B1* mRNA in mouse vagina. (A) *Sema4D* mRNA levels were significantly lower in vaginal tissues from OVX WT mice than in vaginal tissues from sham-operated mice. Based on comparisons between OVX-E2 and OVX-oil mice, OVX-E2 treatment induced a significant increase of *Sema4D* mRNA levels in WT vaginal tissues. The *Sema4D* mRNA variant transcribed in Sema4D−/− mice, but not translated into Sema4D protein exhibits an expression pattern similar to that of wild-type *Sema4D* mRNA. Each value represents the mean ± SEM of 5 mice. *P<0.05, ANOVA. (B) *Plexin-B1* mRNA levels in vaginal tissues from any Sema4D−/− mice group were significantly higher than those in vaginal tissues from any WT mice group. In WT or Sema4D−/− mice, any treatment does not induce any significant alteration of *Plexin-B1* mRNA levels in vaginal tissues. Each value represents the mean ± SEM of 5 mice. *P<0.05, ANOVA.

To examine whether OVX and OVX-E2 treatment affected *Sema4D* or *Plexin-B1* mRNA expression, or both, *Sema4D* and *Plexin-B1* mRNA levels in vaginal tissues were quantified via real-time RT-PCR for sham-operated WT and Sema4D−/− mice, as well as OVX mice, OVX-oil mice and OVX-E2 mice. *Sema4D* mRNA levels in vaginal tissues from WT OVX mice were significantly lower than in vaginal tissues from WT sham-operated mice. Conversely, *Sema4D* mRNA levels were significantly higher in vaginal tissues from OVX-E2 WT mice than vaginal tissues from OVX or OVX-oil mice (Fig. 3A); these findings indicated that estrogen mediated transcriptional modulation of *Sema4D* gene. Real-time PCR with forward and reverse primers corresponding to nucleotide sequences in the second and third exons of the *Sema4D* gene, respectively, detected a *Sema4D* mRNA variant in Sema4D−/− vagina tissues (Fig. 3A). The *Sema4D* mRNA variant did not contain the region transcribed from the coding sequences in the first exon of the *Sema4D* gene because the coding sequences covering the translation-initiation codon are not found in the Sema4D−/− mice genome [21]. Thus, the *Sema4D* mRNA variant was not translated to Sema4D protein. Indeed, the Sema4D protein could not be detected in the Sema4D−/− vagina (Fig. 2B, C) or in Sema4D−/− immune cells [21]. Levels of this mRNA variant were significantly higher in Sema4D−/− vaginal tissues from OVX-E2 mice than in vaginal tissues from OVX or OVX-oil mice (Fig. 3A); these findings indicated that estrogen mediated transcriptional modulation of this *Sema4D* mRNA variant. *Plexin-B1* mRNA levels in vaginal tissues from sham-operated Sema4D−/− mice, as well as OVX, OVX-oil, and OVX-E2 mice, were significantly higher than *Plexin-B1* mRNA levels in any WT group (Fig. 3B). However, neither OVX nor OVX-E2 treatment significantly altered *Plexin-B1* mRNA levels in the vaginal tissues of WT or Sema4D−/− mice (Fig. 3B). Thus, the increase of Plexin-B1 reorganization into an active form in vaginal tissues of OVX-E2 mice resulted from an increase in the estrogen-dependent cleavage of Plexin-B1 precursor.

### Sema4D is Integral for Estrogen-dependent Vaginal Epithelial Apoptosis *In vivo*

**Figure 4:**
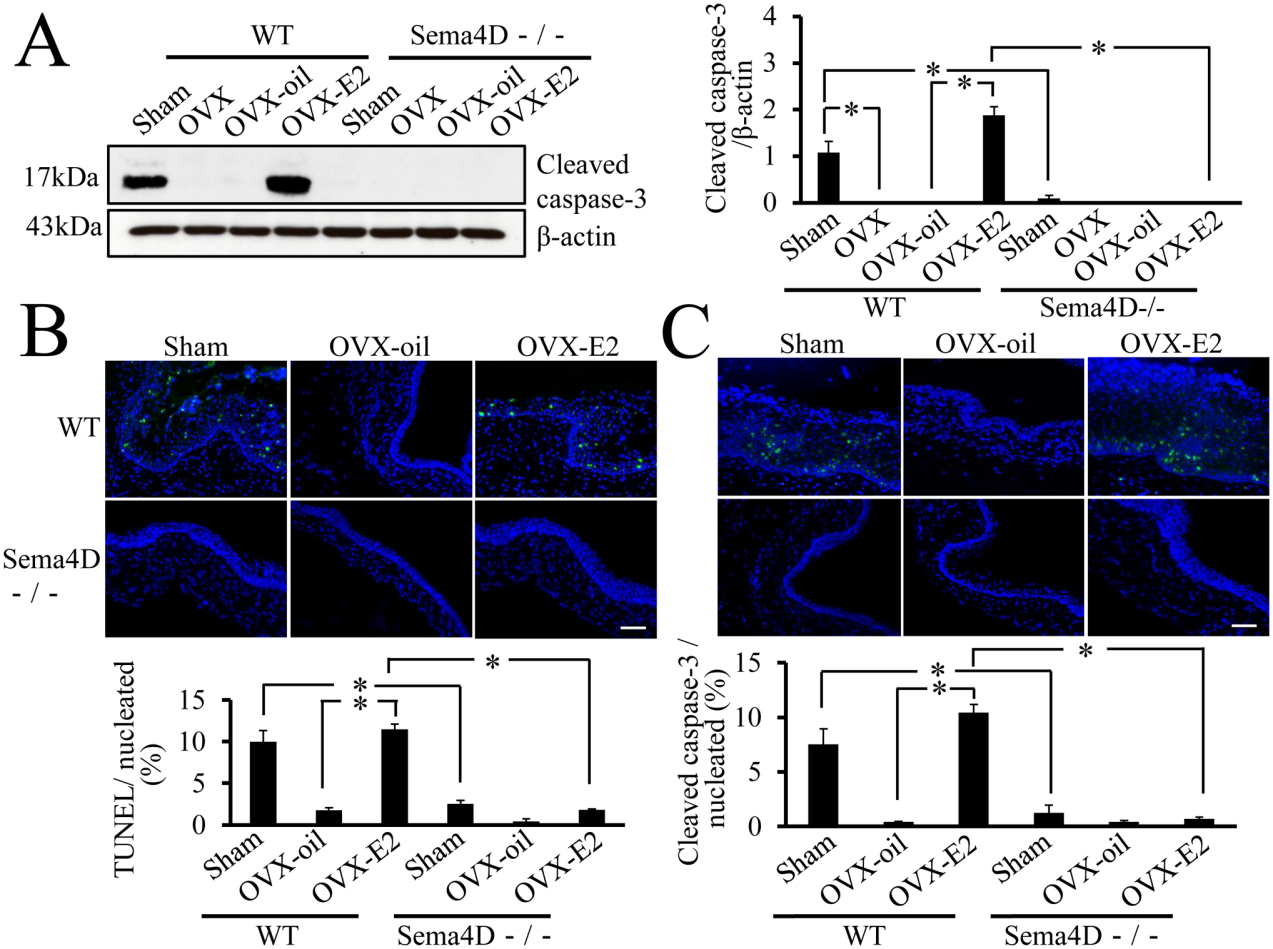
Essential role of Sema4D in estrogen-mediated vaginal apoptosis. (A) Both OVX and OVX-oil treatment of WT (Sema4D+/+) mice result in a significant decrease in cleaved caspase-3 level in vaginal tissue relative to cleaved caspase-3 levels in sham-operated mice (Sham). All vaginal tissue samples were taken from 5-week-old mice. Levels of cleaved caspase-3 were significantly higher in vaginal tissues from WT OVX-E2 mice than in vaginal tissues from WT OVX or WT OVX-oil mice. Levels of cleaved caspase-3 in vaginal tissue did not differ among OVX, OVX-E2, and OVX-oil Sema4D−/− mice; these findings indicate that Sema4D is essential to estrogen-mediated vaginal apoptosis. Each value represents the mean ± SEM of 5 mice. *P<0.05, ANOVA. (B, C) Both TUNEL assays and cleaved caspase-3 immunohistochemistry with vaginal tissue sampled from sham-operated (Sham) 5-week-old WT female (Sema4D+/+) mice show that the number of apoptotic epithelial cells is significantly larger than that in samples from Sham-treated Sema4D−/− mice. OVX-oil treatment of WT mice significantly decreases apoptotic cell number in 5-week-old vaginal epithelia relative to that in Sham; compared with OVX-oil treatment, OVX-E2 treatment of WT mice induces a significant increase in apoptotic cell number in 5-week-old vaginal epithelia, comparable to the level of Sham. OVX-E2 treatment does not induce significant apoptosis in 5-week-old vaginal epithelia of Sema4D−/− mice. Data are shown as means ± SEM; n = 5 per group.

To determine whether Sema4D is essential to vaginal epithelial apoptosis *in vivo*, we measured and compared apoptosis in WT and Sema4D−/− mice; these mice were ovariectomized or sham-operated 3 weeks after birth and then treated with estrogen or vehicle until 5 weeks after birth. Caspase-3 activation was used to measure vaginal apoptosis, and caspase-3 activation was significantly higher in WT OVX-E2 mice than in WT OVX-oil mice (Fig. 4A, C). In contrast, based on both western blot analysis and immunohistochemistry, vaginal apoptosis in ovariectomized Sema4D−/− mice remained low even in Sema4D−/− OVX-E2 mice (Fig. 4A, B, C). The results indicated that Sema4D was essential for estrogen-dependent vaginal epithelial cell apoptosis *in vivo* during the postnatal vaginal tissue remodeling that occurs 5 weeks after birth.

### Dephosphorylation of Akt and ERK at the Time of Vaginal Opening is an Estrogen-dependent, Sema4D-mediated Event in Vaginal Epithelium

**Figure 5:**
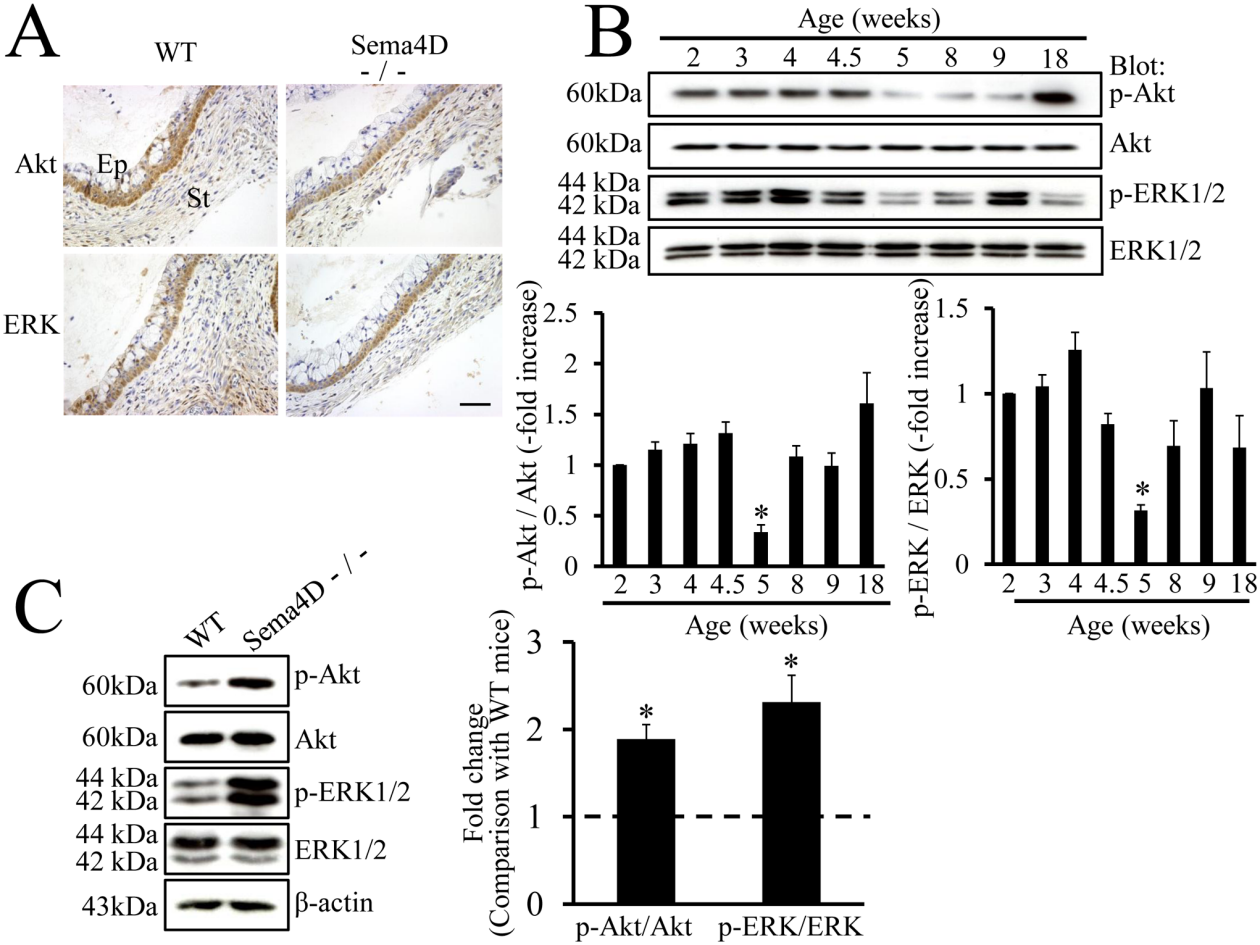
Dephosphorylation of Akt and ERK during vaginal opening. (A) Immunohistochemistry demonstrates that Akt and ERK are expressed in vaginal epithelium of both WT (Sema4D+/+) and Sema4D−/− mice at 5 weeks old at the time of vaginal opening. (B) Western blots show the expression patterns of Akt, ERK, and the respective phosphorylated forms during postnatal vaginal development in WT mice. Both pAkt and pERK levels are lower in samples from 5-week-old mice than in samples from any other stages of development. Age (weeks): vaginal protein extracts from 2-, 3-, 4-, 4.5-, 5-, 8-, 9-, or 18-week-old mice. Each value represents the mean ± SEM of 6 mice. *P<0.05, ANOVA. (C) Western blot analysis reveals significantly higher expression of pAkt and pERK in vaginal tissue samples from 5-week-old Sema4D−/− mice than in vaginal tissue samples from 5-week-old WT (Sema4D+/+) mice. The ratios of pAkt to Akt and separately of pERK to ERK were higher in vaginal tissue samples from 5-week-old Sema4D−/− than in vaginal tissue samples from WT mice. Data are shown as the mean ± SEM; n = 6 per mouse group. *P<0.05, Student’s t-test.

The mechanism suppressing activation of Akt and ERK may operate downstream of semaphorin signaling [16], [23]; therefore, we examined the localization of Akt and ERK in the mouse vagina. Immunohistochemical findings indicated that these molecules were present in vaginal epithelium of WT and of Sema4D−/− mice (Fig. 5A). To examine whether dephosphorylation of Akt and of ERK operates in vaginas of 5-week-old WT mice, we used western blots to measure phosphorylated and unphosphorylated forms of these proteins during mouse vaginal development. The levels of both p-Akt and p-ERK decreased significantly in vaginas of 5-week-old WT mice at the time of vaginal opening; these findings indicated that Sema4D signal suppressed phosphorylation-dependent activation of Akt and of ERK (Fig. 5B).

To examine whether the decreases in p-Akt and in p-ERK depended on Sema4D, we compared WT and Sema4D−/− mice with regard to the levels of p-Akt and p-ERK in vaginal epithelium 5 weeks after birth. Western blot analysis showed that p-Akt and p-ERK expression was significantly higher in the Sema4D−/− vaginal tissue than in WT vaginal tissue (Fig. 5C). Thus, Sema4D signal may induce dephosphorylation of Akt and ERK at the time of vaginal opening.

**Figure 6:**
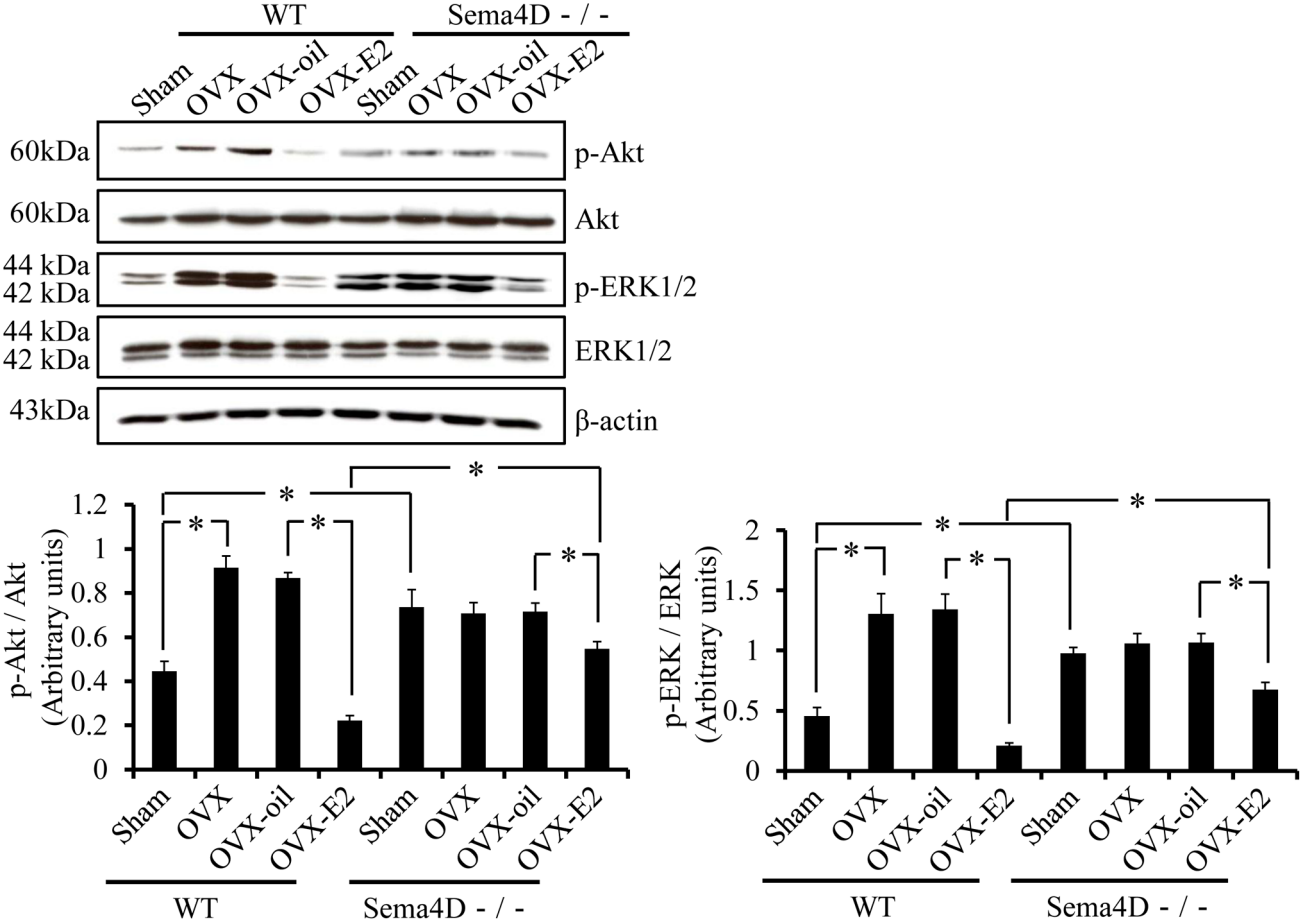
Sema4D contributes to the estrogen-dependent dephosphorylation of Akt and ERK during mouse vaginal opening. Western blot shows that ovariectomy (OVX) and ovariectomy plus oil (OVX-oil) both increase the phosphorylation level of Akt and ERK1/2 in vaginal tissue from 5-week-old WT (Sema4D+/+) mice. β-estradiol (E2) supplementation after ovariectomy (OVX-E2) induces significant dephosphorylation of Akt and ERK1/2 in vaginal tissue from 5-week-old WT mice. OVX and OVX-oil do not significantly increase phosphorylation of Akt and ERK1/2 in vaginal tissue from 5-week-old Sema4D−/− mice relative to that in sham-operated mice (Sham). OVX-E2 induces weak but significant dephosphorylation of Akt and ERK in vaginal tissue from 5-week-old Sema4D−/− mice. Dephosphorylation levels in the Sema4D−/− vagina is significantly lower than that in the WT vagina. Each data point represents the mean ± SEM of 3 to 6 mice. *P<0.05, ANOVA.

To determine whether the decreases in p-Akt and p-ERK expression in mouse vaginal tissue 5 weeks after birth are estrogen dependent and Sema4D mediated, we measured p-Akt and p-ERK levels in vaginal tissues from WT and from Sema4D−/− mice that had been ovariectomized or sham-operated 3 weeks after birth and treated with vehicle only or estrogen thereafter. In vaginal tissue of WT mice, p-Akt and p-ERK expression 5 weeks after birth was significantly higher in OVX mice than in sham-operated mice (Fig. 6). On western blots, p-Akt and p-ERK levels were significantly lower in vaginal tissues from 5-week-old WT OVX-E2 mice than in those from OVX mice or from OVX-oil mice (Fig. 6); these findings indicated that regulation of dephosphorylation of Akt and ERK was estrogen dependent. To determine the extent to which this change in Akt and ERK phosphorylation depended on Sema4D, we examined tissues from Sema4D−/− mice. Notably, for Sema4D−/− mice, phosphorylation levels of Akt and ERK did not differ significantly among vaginal tissues from sham-operated, OVX, and OVX-oil mice (Fig. 6). However, Akt and ERK phosphorylation in vaginal tissue was significantly lower in Sema4D−/− OVX-E2 mice than in Sema4D−/− OVX-oil mice, but the Akt and ERK phosphorylation in Sema4D−/− OVX-E2 mice was significantly higher than that in WT OVX-E2 mice (Fig. 6). These results indicated that normal estrogen-dependent dephosphorylation of Akt and ERK at the time of vaginal opening was partially, but not completely, dependent on Sema4D.

## Discussion

Using Sema4D−/− BALB/c mice, we documented three novel, crucial observations regarding the estrogen-dependent apoptosis that occurs during postnatal vaginal opening in mice. We found that 1) Sema4D, which is classified as a class 4 semaphorin, played an indispensable role as a downstream effector of estrogen action during apoptosis of vaginal epithelial cells as the vagina opens; 2) estrogen-dependent Sema4D processing and estrogen-dependent Plexin-B1 reorganization increased Sema4D signal transduction efficiency during vaginal opening; and 3) Sema4D contributed to estrogen-dependent attenuation of both Akt and ERK signaling during vaginal opening.

The high incidence of imperforate vagina and the prominent decrease in vaginal epithelial apoptosis during puberty in Sema4D−/− mice led to the hypothesis that Sema4D could induce apoptosis of vaginal epithelial cells. Our previous findings regarding Sema4D−/− vaginal epithelial cells in culture demonstrate the apoptosis-inducing activity of Sema4D [7]. Similarly, Sema3A, a member of class 3 semaphorins, induces apoptosis of kidney podocytes [24]. Notably, Sema4D could promote apoptosis of oligodendrocytes to control the differentiation of oligodendrocytes [25]. Furthermore, Sema3A has been implicated in Fas-mediated apoptosis; specifically, it may help the Fas molecule migrate into lipid rafts [26]. Thus, semaphorins exhibit crucial roles not only in axon guidance, but also in induction of apoptosis during development. However, the receptor and signal transduction machinery involved in semaphorin-mediated apoptosis have not been explored in detail. Our previous findings indicate that Plexin-B1 is involved in Sema4D-induced apoptosis of vaginal epithelial cells in culture [7]. In WT ovariectomized mice, apoptosis in vaginal epithelia was significantly higher in the OVX-E2 mice than in vehicle-treated ovariectomized mice (Fig. 4). In contrast, OVX-E2 treatment did not induce significant apoptosis in 5-week-old vaginal epithelia of Sema4D−/− mice (Fig. 4). Thus, these data indicated that Sema4D functioned as an essential downstream effector of estrogen action mediating vaginal epithelial apoptosis *in vivo* during postnatal tissue remodeling. Thus, this is the first manuscript reporting that a semaphorin known as an axon guidance molecule exhibited a decisive role in the downstream effects of estrogen action in the process of mouse vaginal opening [1]. Our study of ovariectomized mice further revealed that Sema4D was involved in the estrogen-dependent dephosphorylation of pAkt and pERK in postnatal vaginal tissue remodeling (Fig. 6). Similar to the signal transduction pathway of Sema4D that functions in neuronal growth cone guidance [14], [15], [16], the Sema4D pathway in vaginal epithelium may involve dephosphorylation of pAkt and pERK; this dephosphorylation may depend on downregulation of Ras family members; this Ras family downregulation may in turn be caused by Plexin-B1 GAP activities that are activated by Sema4D signal in vaginal epithelial apoptosis during vaginal opening in mice.

Conversion of membrane-bound Sema4D to secreted Sema4D was significantly higher during vaginal opening than during any other period of development (Fig. 1A, B, C, D). The increase in the conversion of membrane-bound Sema4D to secreted Sema4D may facilitate induction of apoptosis by increasing the activity of Sema4D as a ligand that acts on both neighboring and distant cells [19]. Sema4D mRNA levels peaked in vaginal epithelium 5 weeks after birth (Fig. 1E); this increase may have boosted apoptosis by increasing ligand quantity. Based on comparisons between OVX-E2 and OVX-oil mice, OVX-E2 treatment induced a significant increase in *Sema4D* mRNA levels in mouse vagina (Fig. 3A), indicating that estrogen may have modulated transcription of the *Sema4D* gene. Since the ratio of soluble Sema4D to total Sema4D increased in concert with the increase of *Sema4D* mRNA in vaginal tissues of WT OVX-E2 mice (Fig. 2B, C), the significant increase in soluble Sema4D in OVX-E2 mice relative to that in OVX-oil mice resulted from enhanced estrogen-dependent cleavage of membrane-bound Sema4D to soluble Sema4D in vaginal tissues in OVX-E2 mice relative to that in OVX-oil mice. Accordingly, experiments involving ovariectomy demonstrated that the conversion from membrane-bound to secreted Sema4D was estrogen dependent (Fig. 2B, C); therefore, the activities and/or amounts of cleavage enzyme releasing soluble Sema4D by cutting membrane-bound Sema4D may increase in response to the estrogen increase at the time of vaginal opening. Although a previous study reported that the function of the cleavage enzyme is to produce the secreted form of Sema4D by cutting membrane-bound Sema4D during T cell activation in the immune system [19], the present study is the first to demonstrate the estrogen-dependent conversion of Sema4D from a membrane-bound precursor to a secreted protein.

Expression of a short fragment of Plexin-B1 that results from enzyme-dependent proteolysis of a Plexin-B1 precursor peaked at the time of vaginal opening; this pattern was similar to the pattern of soluble Sema4D production (Fig. 1A, F). Research with human cell lines showed that a proprotein convertase cleaves the precursor of Plexin-B1 and results in a conformational change of the Plexin-B1 molecule and enhancement of Plexin-B1-mediated signal transduction [20]. Sites that contain the amino acid sequence RXXR and that may be recognized by proprotein convertases actually reside in the mouse Plexin-B1 molecule (R_546_EERR and R_1169_GPR, Figure S2). The predicted molecular size of the cleavage Plexin-B1 molecule produced by proprotein convertases coincides with the size actually detected on western blots probe with anti-Plexin-B1 antibodies (Fig. 1A, Figure S2). The activated form of Plexin-B1, structurally modified and activated for signaling by the proteolytic cleavage, increased most in amount at the time of vaginal opening; this proteolytic conversion may have accelerated vaginal epithelial apoptosis by enhancing signal transduction of Sema4D. Conversion of Plexin-B1 precursor into active Plexin-B1 in mouse vaginal tissue was estrogen dependent (Fig. 1A, Fig. 2B, D); therefore, the amounts and/or activities of the proprotein convertases that cleave Plexin-B1 may also increase in an estrogen-dependent manner. Even though the receptor activation that is mediated by the proteolytic cleavage of Plexin-B1 was previously observed in several cultured cell lines [20], our study is the first to demonstrate the estrogen dependency of the proteolytic conversion of Plexin-B1 during mouse vaginal opening.

C57BL/6 Sema4D−/− mice do not exhibit a high incidence of imperforate vagina, but BALB/c Sema4D−/− mice do. LH-RH neuron precursor cells in C57BL/6 Sema4D−/− mice exhibit defective migration from the olfactory placode to the hypothalamus during embryonic brain development [27]. A significant decrease in secondary ovarian follicles is also evident in Sema4D−/− C57BL/6 mice [28]. Our previous study revealed no significant difference in serum estrogen levels between Sema4D−/− BALB/c mice and WT mice 5 weeks after birth. [7]. Since injection of β-estradiol into infant Sema4D−/− mice does not lead to vaginal opening, the possibility that vaginal atresia in pubescent Sema4D−/− mice was caused by insufficient secretion of estrogen was excluded [7]. Further experiments involving ovariectomy, estrogen supplementation, and WT and Sema4D−/− BALB/c mice clearly revealed that the Sema4D was essential to vaginal epithelial apoptosis, which was regulated by the estrogen present during vaginal opening (Fig. 4A, B, C). Previous findings indicate that Plexin-B1 is the receptor that induces apoptosis of vaginal epithelial cells in culture [7]; however, a closed vaginal phenotype has not been reported in Plexin-B1−/− C57BL/6 mice [29], [30]. Future experiments may be necessary to examine whether the closed vaginal phenotype also occurs in BALB/c mice lacking Plexin-B1. The structural conversion of Sema4D and of Plexin-B1 that was evident in BALB/c mice was also evident in C57BL/6 mice during postnatal vaginal development (Fig. S3). However, the day of vaginal opening (5 weeks old) coincided with the peak in the conversion of both Sema4D and Plexin-B1 in BALB/c mice, (Figure 1), but there was no concordance between the day of vaginal opening (37.18±0.787 day old, n = 17) and the peak of the proteolytic conversions (24 to 28 days old) in C57BL/6 mice (Fig. S3). These observations may illustrate that postnatal vaginal tissue remodeling processes are more dependent on Sema4D/Plexin-B1 signal transduction system in BALB/c mice than in C57BL/6 mice.

Thus, we propose that extensive apoptosis that occurs in epithelial cells in the vaginal cavity during postnatal vaginal opening in BALB/c mice is induced by Sema4D–Plexin-B1 signaling and that this signaling is enhanced by the estrogen-dependent increase in both soluble Sema4D and active Plexin-B1 5 weeks after birth.

## Supporting Information

Figure S1 **Confirmation of the effect of ovariectomy and estrogen supplementation.** Both ovariectomized (OVX) WT (Sema4D+/+) and Sema4D−/− mice exhibit a significant decrease in uterine weight when compared with sham-operated animals. WT and Sema4D−/− mice with ovariectomy and estrogen supplementation (OVX-E2) exhibit significant increases in uterine weight when compared with ovariectomized mice supplemented with oil (OVX-oil). Values shown are mean ± SEM. *P<0.05. OVX, ovariectomy; OVX-oil, ovariectomy plus oil supplementation; OVX-E2, ovariectomy plus 17β-estradiol supplementation.
(TIF)

Figure S2 **The sites of Plexin-B1 cleaved by proprotein convertase.** Plexin-B1, the Sema4D receptor, transforms from a precursor protein into an active protein via a conformational change that is caused by proprotein convertase-dependent cleavage of Plexin-B1. The proprotein convertase recognizes and cleaves the RXXR sequence residing in the target protein. A protein fragment presumed to result from the cleavage of R_546_EERR and R_1169_GPR of mouse Plexin-B1 is detected as a 75 kDa band on the western blot probed with anti-Plexin-B1 antibody (A-8). A-8 is a monoclonal antibody raised against the peptide fragment comprising amino acids 771-1070 of human plexin-B1, which is highly conserved with mouse plexin-B1 and covered by the 75 kDa region detected by western blot with A8. A previous study showed that the region covering the transmembrane and intracellular region, which is generated by proprotein convertase-mediated digestion of mouse plexin-B1, has a molecular size of 150 kDa [30]. EC: extracellular domain, TM: transmembrane domain, IC: intracellular domain, PCs: proprotein convertases.
(TIF)

Figure S3 **Conversion of Sema4D and Plexin-B1 into respective soluble and active form during C57BL/6 vaginal development.** The conversion of both Sema4D and Plexin-B1 into the respective soluble and active forms peaked 3.5 to 4 weeks (24 to 28 days) after birth; this time period does not coincide with the vaginal opening in C57BL/6 mice (37 days old).
(TIF)
