# Supplementary figures and images for: Estrogen-Dependent Proteolytic Cleavage of Semaphorin 4D and Plexin-B1 Enhances Semaphorin 4D-Induced Apoptosis during Postnatal Vaginal Remodeling in Pubescent Mice
Source: PLoS One. 2014 May 19;9(5):e97909. doi: 10.1371/journal.pone.0097909 (PMC4026538; doi:10.1371/journal.pone.0097909)

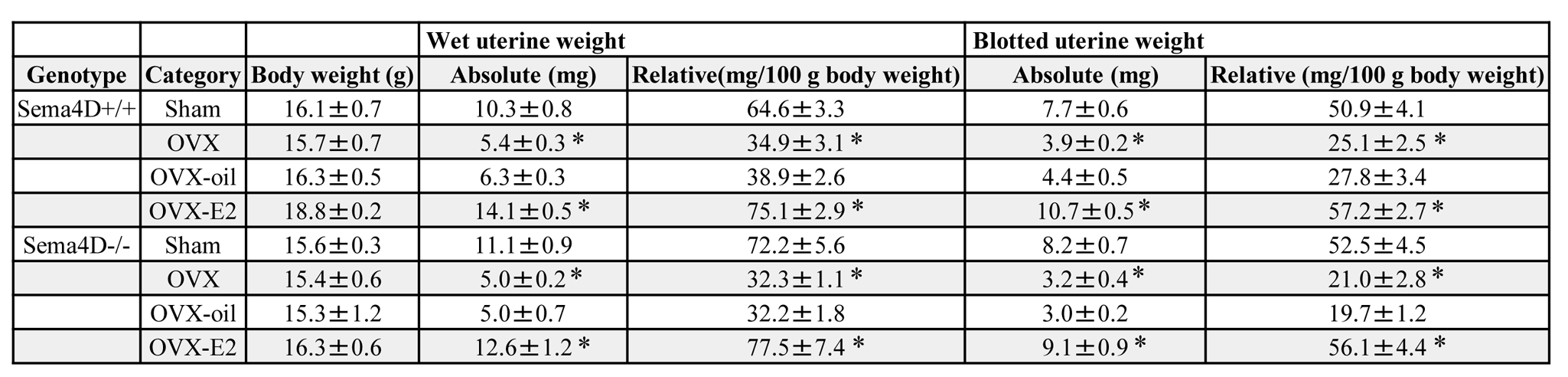

Supplement: Figure S1 — Confirmation of the effect of ovariectomy and estrogen supplementation. Both ovariectomized (OVX) WT (Sema4D+/+) and Sema4D−/− mice exhibit a significant decrease in uterine weight when compared with sham-operated animals. WT and Sema4D−/− mice with ovariectomy and estrogen supplementation (OVX-E2) exhibit significant increases in uterine weight when compared with ovariectomized mice supplemented with oil (OVX-oil). Values shown are mean ± SEM. *P<0.05. OVX, ovariectomy; OVX-oil, ovariectomy plus oil supplementation; OVX-E2, ovariectomy plus 17β-estradiol supplementation. (TIF) [file pone.0097909.s001.tif]

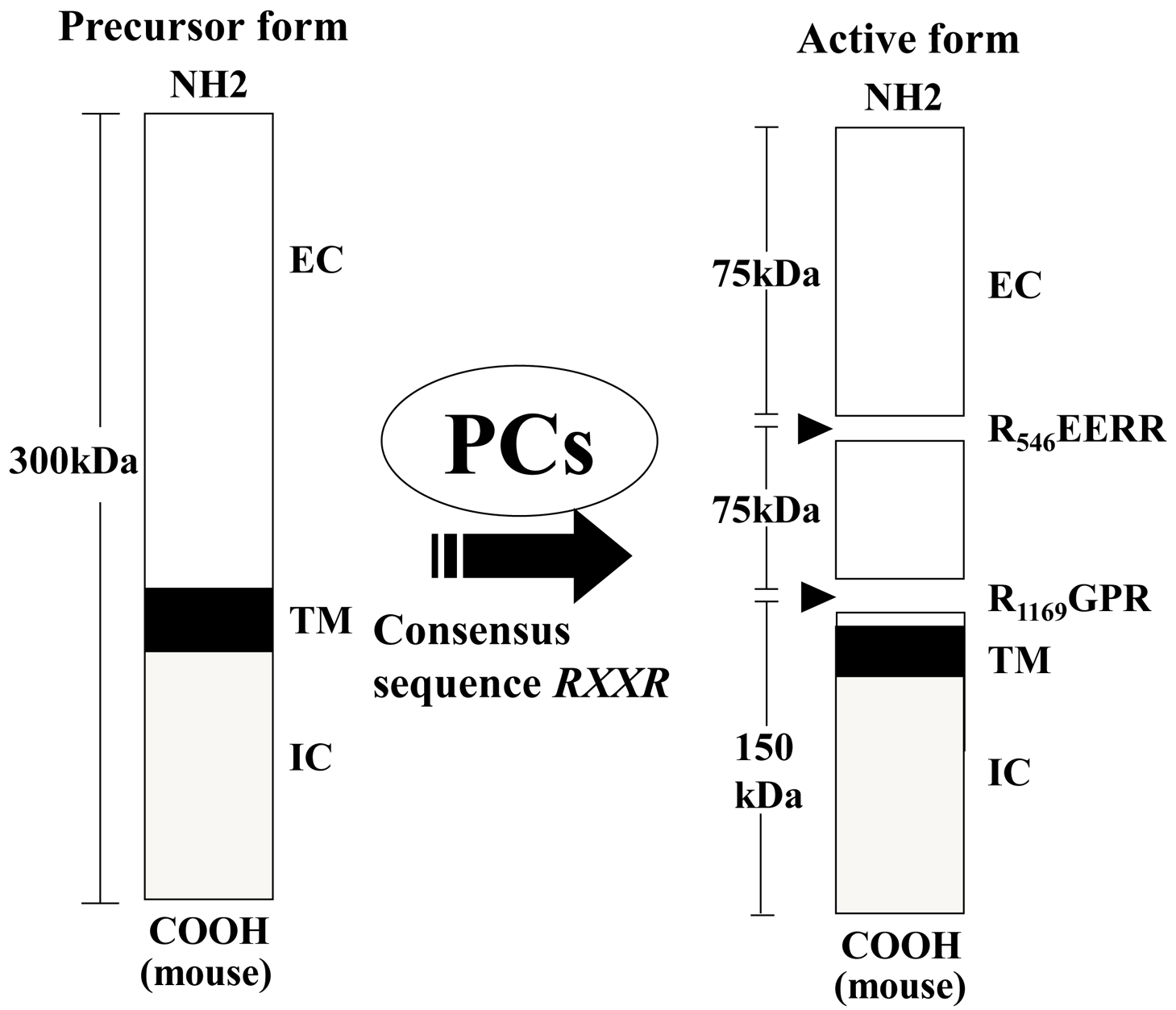

Supplement: Figure S2 — The sites of Plexin-B1 cleaved by proprotein convertase. Plexin-B1, the Sema4D receptor, transforms from a precursor protein into an active protein via a conformational change that is caused by proprotein convertase-dependent cleavage of Plexin-B1. The proprotein convertase recognizes and cleaves the RXXR sequence residing in the target protein. A protein fragment presumed to result from the cleavage of R546EERR and R1169GPR of mouse Plexin-B1 is detected as a 75 kDa band on the western blot probed with anti-Plexin-B1 antibody (A-8). A-8 is a monoclonal antibody raised against the peptide fragment comprising amino acids 771-1070 of human plexin-B1, which is highly conserved with mouse plexin-B1 and covered by the 75 kDa region detected by western blot with A8. A previous study showed that the region covering the transmembrane and intracellular region, which is generated by proprotein convertase-mediated digestion of mouse plexin-B1, has a molecular size of 150 kDa [30]. EC: extracellular domain, TM: transmembrane domain, IC: intracellular domain, PCs: proprotein convertases. (TIF) [file pone.0097909.s002.tif]

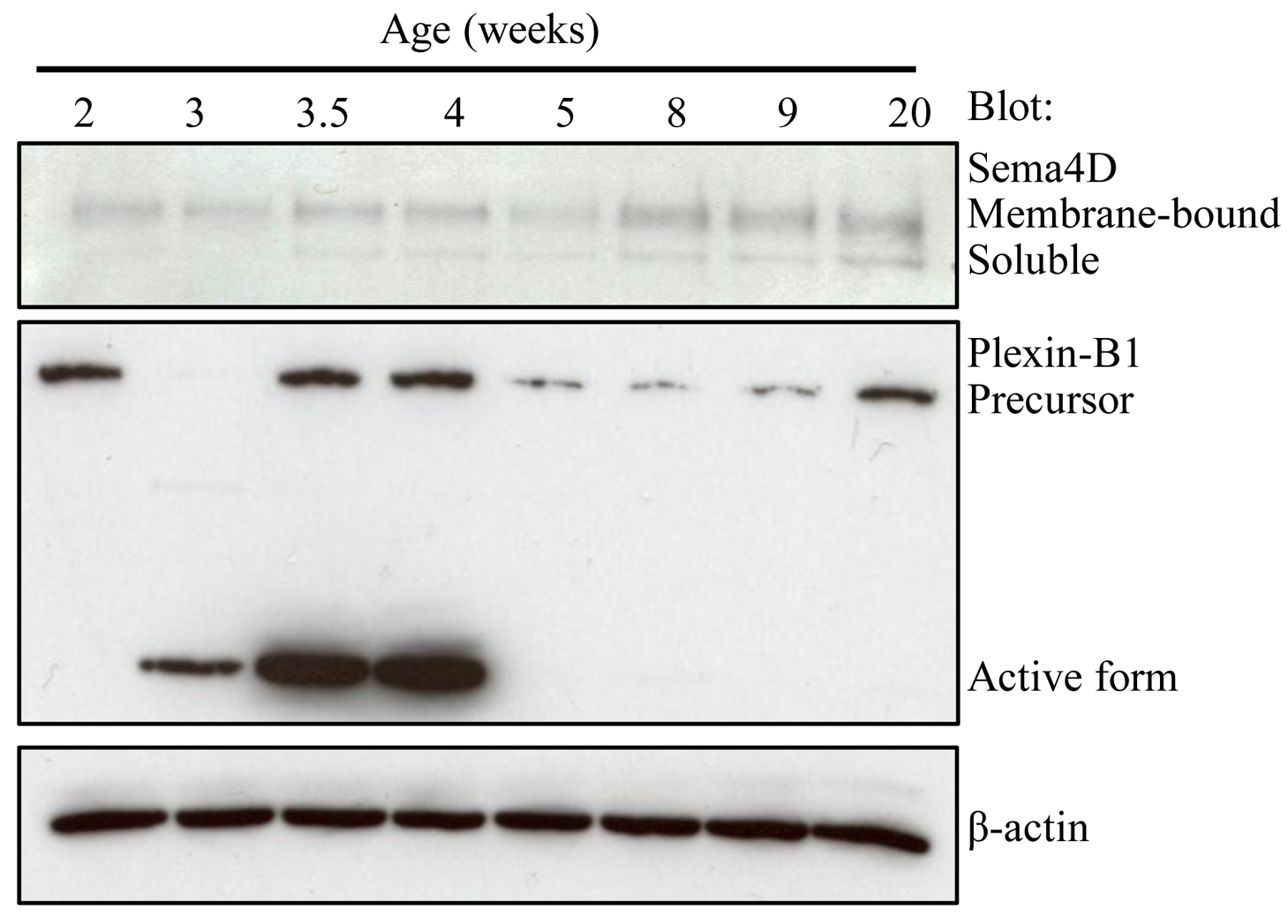

Supplement: Figure S3 — Conversion of Sema4D and Plexin-B1 into respective soluble and active form during C57BL/6 vaginal development. The conversion of both Sema4D and Plexin-B1 into the respective soluble and active forms peaked 3.5 to 4 weeks (24 to 28 days) after birth; this time period does not coincide with the vaginal opening in C57BL/6 mice (37 days old). (TIF) [file pone.0097909.s003.tif]
